# The Apical Endocytic-Lysosomal Apparatus in CLCN5 Mutations with Phenotypic-Genotypic Correlations in Three Cases

**DOI:** 10.3390/ijms25020966

**Published:** 2024-01-12

**Authors:** Tibor Kalmár, Dániel Jakab, Zoltán Maróti, Orsolya Lakatos, Tibor Vas, Csaba Bereczki, Béla Iványi

**Affiliations:** 1 Department of Pediatrics, Albert Szent-Györgyi Medical School and Health Center, University of Szeged, 6720 Szeged, Hungary; jakab.daniel@med.u-szeged.hu (D.J.); maroti.zoltan@med.u-szeged.hu (Z.M.); bereczki.csaba@med.u-szeged.hu (C.B.); 2 Department of Pediatrics, University of Pécs, 7624 Pécs, Hungary; lakatos.orsolya@pte.hu; 3 Department of Internal Medicine, University of Pécs, 7624 Pécs, Hungary; vas.tibor2@pte.hu; 4 Institute of Pathology, Albert Szent-Györgyi Medical School and Health Center, University of Szeged, 6720 Szeged, Hungary; ivanyi.bela@med.u-szeged.hu

**Keywords:** apical endocytic apparatus, CLCN5 mutations, low-molecular-weight proteinuria, lysosomes, mitochondria, receptor-mediated endocytosis, Dent disease

## Abstract

Dent disease type 1 is characterized by pathogenic *CLCN5* gene variants and impaired receptor-mediated endocytosis in proximal tubules. However, mutation-related abnormalities in proximal tubules have not yet been described. Here, we present three patients with CLCN5 alterations and distinct morphological changes of the apical endocytic-lysosomal apparatus. The proximal tubular ultrastructure was investigated in kidney biopsy samples of three boys genotyped for non-nephrotic proteinuria. Controls: seven patients with nephrotic-range glomerular proteinuria. The genotyping findings revealed an already-known missense mutation in one patient and hitherto undescribed frameshift variants in two patients. Low-molecular-weight proteinuria, focal global glomerulosclerosis, proximal tubular changes, and tubular calcium deposits characterized each case. Three subsets of proximal tubular cells were observed: those without any abnormality, those with aplasia of apical endocytic-lysosomal apparatus and shrinkage of cells, and those with hypoplasia of apical endocytic apparatus, accumulation of proteinaceous substance in dysmorphic lysosomes, and dysmorphic mitochondria. The distribution of subsets varied from patient to patient. In one patient with a frameshift variant, an oxidative stress-like injury of proximal tubular cells and podocytes accompanied the above-mentioned alterations. Focal aplasia/hypoplasia of apical endocytic apparatus and subsequent changes in cytoplasmic organelles characterized proximal tubules in the *CLCN5* pathogenic variants.

## 1. Introduction

The proximal tubules (PTs) reabsorb 80% of the filtrate that passes through the glomeruli. Low-molecular-weight (LMW) proteins, albumin, and essential nutrients are taken up from the tubular fluid via receptor-mediated endocytosis. The ligands bind to the multiligand receptors megalin and cubilin/amnionless expressed in clathrin-coated pits at the base of microvilli, and then the complexes are internalized into small apical vacuoles that fuse with large apical vacuoles. The vacuoles are acidified by the action of vacuolar H+-ATPase [1,2]. The electrogenic Cl-/H+exchanger ClC-5 protein colocalizes with the H+-ATPase and contributes to endosomal acidification. An acidic pH in vacuoles is necessary for the dissociation of receptors and ligands and the transport of megalin and cubilin to the apical membrane through dense apical tubules. Once internalized into endosomes, LMW proteins are mainly transported to lysosomes for degradation [3]. The energy required for the processes involved in reabsorption, intracellular transport and the lysosomal work-up of endocytosed substances is generated by the abundant mitochondria, working in cooperation with peroxisomes. The latter organelle has a role in the elimination of free oxygen radicals.

The ClC-5 protein, encoded by the *CLCN5* gene located on chromosome Xp11.23, is expressed in the endosomes of PT cells, and to a lesser extent in cells of the thick ascending limb of Henle’s loop, alpha-intercalated cells of collecting ducts, podocytes, and parietal epithelial cells [4]. Loss-of-function mutations in the *CLCN5* gene lead to Dent disease type 1 (DD1). Over 250 pathogenic variants of the *CLCN5* gene have been described [5,6]. In renal biopsies of patients carrying the stop codon or missense mutations, the immunohistochemical expression of ClC-5 protein was downregulated in both tubular and glomerular compartments [7,8], and the apical expression of endocytic machinery proteins in PTs was found to be markedly decreased or irregular [9]. The pathogenic variants of the *CLCN5* gene impair receptor-mediated endocytosis and intracellular transport in PTs [7,10,11], and alter the function of podocytes [8].

Clinically, DD1 is an X-linked recessive renal tubulopathy, characterized by an onset in childhood, LMW proteinuria (a marker of defective receptor-mediated endocytosis), hypercalciuria, medullary nephrocalcinosis/kidney stones, and the slow progression to end-stage kidney disease in adulthood. Proximal tubular defects of differing severity are common [4,6]. Proteinuria and microhematuria are frequently observed, the proteinuria being sometimes in the nephrotic range [6]. The evaluation of renal biopsies revealed focal global glomerulosclerosis (FGGS) with or without segmental glomerulosclerosis, mild segmental foot process effacement, a varying degree of interstitial fibrosis and tubular atrophy, interstitial lymphocytic infiltrates, non-specific tubular damage, and the deposition of calcium phosphate crystals [6]. There were cases, however, which did not show any abnormality, suggesting phenotypic heterogeneity of DD1 [4,10]. The publications on altogether 59 patients with DD1 and the electron microscopical evaluation of kidney biopsy samples had no reported alterations in proximal tubular cells attributed to the mutation [1,8,10,12,13,14,15,16,17,18,19,20,21,22] (see Table 1), which suggested DD1 was a functional tubulopathy. Except for two publications [1,16], however, the focus was on identifying alterations of podocytes and the glomerular basement membrane (GBM), and therefore proximal tubular changes related to pathogenic *CLCN5* variants might have gone undetected or been overlooked.

Here, we present the renal biopsy findings of three unrelated boys evaluated for non-nephrotic proteinuria in whom the genotyping performed after the biopsy procedure demonstrated three different pathogenic *CLCN5* variants, and the clinical search for LMW proteinuria proves positive. Then, the electron microscopical and histological appearance of apical endocytic apparatus (AEA) in our Dent patients was reinvestigated and compared with controls representing stimulated receptor-mediated endocytosis of proximal tubules, i.e., patients with heavy glomerular proteinuria. The approach allowed us to identify and describe here for the first time the hypoplasia or even absence of the AEA in Dent patients as a manifestation of defective receptor-mediated endocytosis. The abnormality unevenly affected the proximal tubules, and it was associated with profound changes in organelles of proximal tubular epithelial cells.

## 2. Case Presentation

### 2.1. Control Patients with Heavy Proteinuria

**Figure 1:**
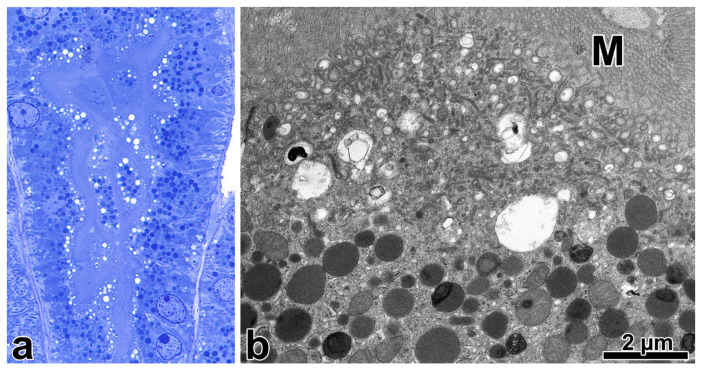
The apical endocytic-lysosomal apparatus from a case of minimal change nephropathy. (**a**) The numerous and evenly distributed endocytic vacuoles beneath the lightly stained brush border form a distinct layer. The lysosomes appear as dark-blue cytoplasmic granules in the apical and mid-portion of cells (toluidine blue, resin-embedded sample, ×100). (**b**) Beneath the microvilli (M), there is a wide zone of apical endocytic apparatus consisting of a great number of small vacuoles and apical dense tubules, and several large vacuoles. The underlying lysosomes are rounded and have a homogeneous electron-dense matrix or contain membranous structures or dark electron-dense aggregates in a moderately electron-dense matrix. The mitochondria have a pale matrix and lamellar cristae, oriented in parallel.

The tubular epithelial cells frequently contained fine cytoplasmic granules of protein resorption droplets in paraffin sections. A prominent layer of apical vacuoles and numerous toluidine blue positive granules underneath the vacuoles were observed in the corresponding semithin sections (Figure 1a). Electron microscopically, a wide zone of AEA, and large number of similar-sized lysosomes often having dense substances in various stages of degradation were seen; and the mitochondria appeared normal (Figure 1b). Peroxisomes with marginal crystalloid were infrequent.

### 2.2. Patients with Dent Disease Type 1 (DD1)

A synopsis of clinical features and renal biopsy findings is shown in Table 2 and Table 3.

#### 2.2.1. Patient 1

**Figure 2:**
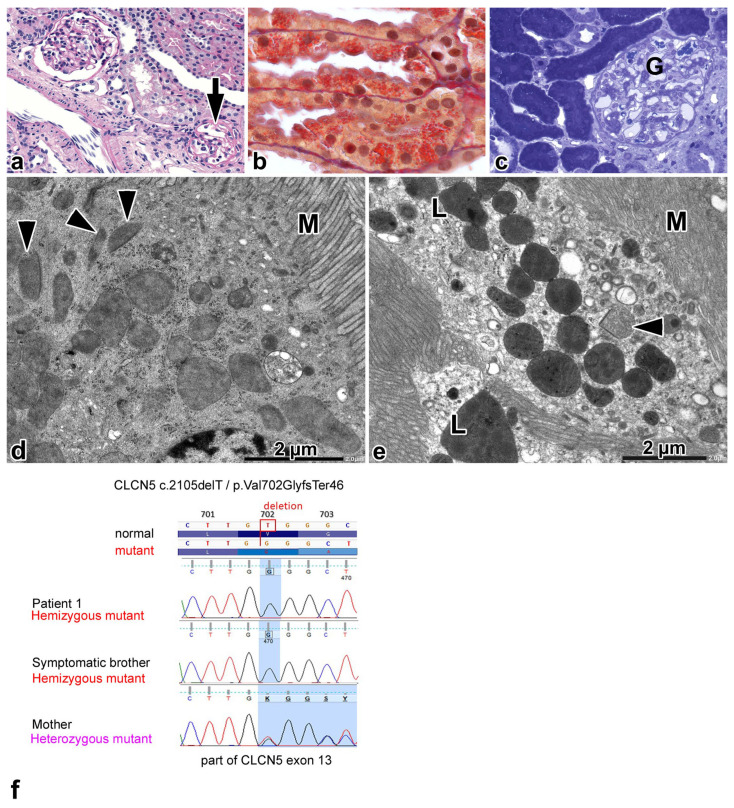
Renal biopsy alterations and the genotyping results for patient 1. (**a**) An apparently normal glomerulus and a glomerulus with segmental collapse and sclerosis of capillary loops (arrow). Periodic acid-Schiff (PAS), ×20. (**b**) The cells of proximal straight tubules are packed with trichrome-positive (red) granules. Acid fuchsin orange G, ×100. (**c**) Proximal convoluted tubular profiles without any discernible apical endocytic-lysosomal apparatus. The staining of the cytosol is homogeneous and dark. The apical part of one proximal tubular cell is shown in Figure 2d. G—glomerulus. Toluidine blue, ×40. (**d**) The apical endocytic-lysosomal apparatus is rudimentary, only a few apical vacuoles and apical dense tubules are discernable, and lysosomes cannot be identified. The matrix and cristae of mitochondria have not separated from each other. M—microvilli, arrowhead—peroxisome with crystalloid. (**e**) Proximal tubular cells with a thinned zone of apical endocytic apparatus. The lysosomes have an irregular contour and dense matrix, and they are filled with markedly electron-dense aggregates. The mitochondria have a dense matrix, and the cristae are sparse and have curved outlines. M—microvilli, arrowhead—peroxisome with crystalloid. (**f**) The symptomatic boys and their mother exhibit deletion in exon 13 of the *CLCN5* gene.

A 1.5-year-old boy was evaluated for microhematuria, and he was subsequently monitored for 14 years. A kidney biopsy was performed at age 6.5 years; and the genetic test concluded the diagnosis at age 12. A light microscopic examination revealed twenty-two glomeruli, three of which were globally sclerotic, and one displayed segmental sclerosis (Figure 2a). Cytoplasmic granules had accumulated in 20% of PT profiles, predominantly the straight segments. The granules stained red with acid fuchsin orange G (Figure 2b), and they did not stain with methenamine silver, and they had no autofluorescence. Eosinophilic casts were noted occasionally in medullary collecting ducts. A few intratubular and interstitial calcified deposits were observed in the medullary portion of the specimen. There were two normal-looking glomeruli in the semithin sections. Several proximal convoluted tubular profiles were devoid of endocytic vacuoles; the staining of cytoplasm was dark and homogeneous, and it was difficult to discern the cell organelles (Figure 2c). Other profiles had apical vacuoles, although low in number. The staining of cytoplasm was lighter, and the cells were packed with cytoplasmic granules, found to be trichrome-positive in the paraffin sections. Electron microscopically, the GBM was normal, and the foot processes were effaced segmentally. The cytoplasm of podocytes had well-developed Golgi apparatus, cystically dilated rough endoplasmic reticulum profiles, a few lysosomes, and normal-looking mitochondria. The brush border microvilli appeared normal. In PT profiles with no apical vacuoles in semithin sections, the AEA was rudimentary, just consisting of a few endocytic vacuoles and apical dense tubules. Lysosomes were rarely seen, and the mitochondria had a homogeneous appearance, and it was difficult to distinguish their matrix from the cristae (Figure 2d). Peroxisomes with crystalloid were frequent. In PT profiles with apical vacuoles and cytoplasmic granules in semithin sections, a narrow zone of AEA was observed. The underlying lysosomes were numerous and contained homogeneous electron-dense aggregates against a slightly paler matrix and/or remnants of cell organelles. Residual bodies were not seen. The mitochondria had different shapes and they had an electron-dense configuration (Figure 2e). Peroxisomes with crystalloid were frequent. The cells of distal nephron segments did not have any abnormalities.

The Sanger sequence analysis (Figure 2f) identified a single nucleotide deletion in exon 13 (*CLCN5* c.2105delT/p.Val702GlyfsTer46) in hemizygous form in the two brothers. The detected variant is absent in the gnomAD, Public HGMD, and ClinVar database. A bioinformatical prediction supported pathogenicity since the deletion of a single nucleotide might have caused a frameshift and premature STOP codon. The predicted length of the truncated mutant protein was 746 (vs full 818) AA length. The boys inherited this variant from their heterozygous carrier mother.

#### 2.2.2. Patient 2

A 13.5-year-old boy was evaluated for accidentally detected proteinuria and chronic kidney disease stage 2. The clinical follow-up revealed intermittent Ca-oxalate crystalluria. Besides kidney disease, the boy had concurrent strabism and mild bilateral conductive hearing loss.

**Figure 3:**
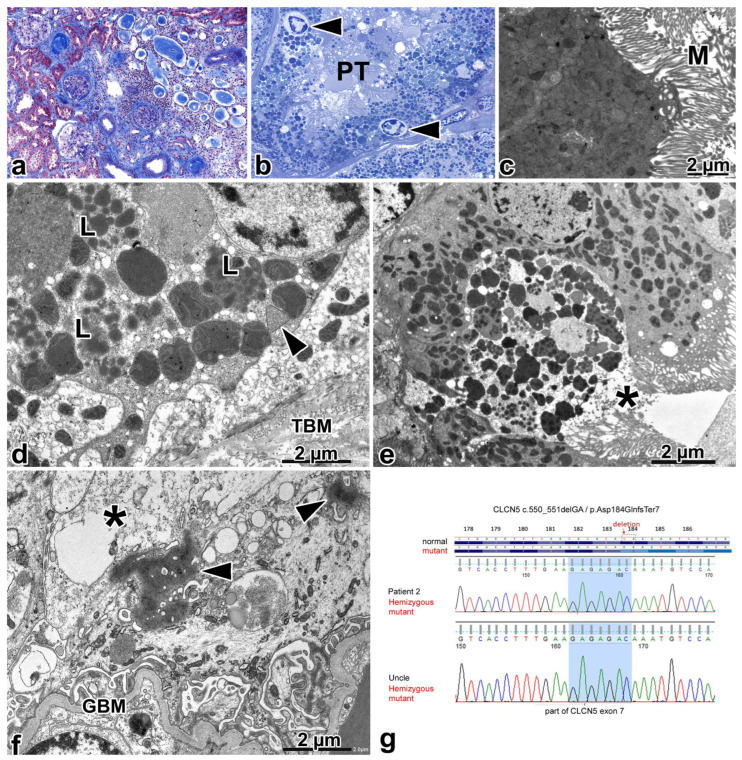
Renal biopsy alterations and the genotyping results for patient 2. (**a**) Patent glomeruli, globally sclerotic glomeruli, and glomeruli with periglomerular fibrosis adjacent to chronic tubulointerstitial injury displaying tubular microcystic change, luminal casts, and mononuclear interstitial infiltrate. Masson’s trichrome, ×10. (**b**) The cells in proximal tubular profile (PT) are packed with cytoplasmic granules having different staining characteristics. The nuclei have condensed chromatin, surrounded by a clear halo (arrowhead). Toluidine blue, ×100. (**c**) The apical part of a proximal tubular cell. The components of apical endocytic apparatus are absent, the cytosol is dense, and it is difficult to discern the few small lysosomes from the mitochondria. M—microvilli. (**d**) The basal region of proximal tubular cell that had a thinned zone of apical endocytic apparatus (not shown). The cytoplasm is packed with different-sized lysosomes (L) that frequently contained electron-dense aggregates. Several lysosomes just have a focally discernable membrane. The mitochondria are dysmorphic, their matrix is hyperdense, and the cristae have curved outlines. Arrowhead—peroxisome with crystalloids. TBM—tubular basement membrane. (**e**) Proximal tubular cells with discernable apical endocytic apparatus. Different-sized lysosomes have accumulated throughout the entire thickness of cells, containing electron-dense aggregates, or membrane-bound structures. The mitochondria are dysmorphic. The rupture of the apical cell membrane can be seen near the asterisk. (**f**) The cytoplasm of a podocyte. Membrane-bound vacuoles, containing degrading material, and dense aggregation of cytoplasm (arrow) can be seen in the cell body. At the top of image, the focal rupture of cell membrane and swelling of the cytosol have occurred (asterisk). The foot processes are segmentally effaced. The glomerular basement membrane (GBM) appears normal. (**g**) Deletion in exon 7 of the *CLCN5* gene has affected the boy and the brother of his maternal grandfather.

In tests, 63 patent glomeruli, 32 globally sclerosed glomeruli, and conspicuous interstitial fibrosis and tubular atrophy of the cortex were observed light microscopically. The sclerotic glomeruli were localized in areas of interstitial fibrosis and tubular atrophy. On the borders of these areas, the glomeruli exhibited periglomerular fibrosis. Further on from these, some of the glomeruli appeared enlarged; the mean glomerular diameter was 190 µm (in the range 166 µm to 230 µm; measured in 8 glomeruli with the vascular pole). Also, the glomerular capillary loops and the GBM showed no alteration. In areas of interstitial fibrosis, there were groups of atrophic tubules that were lined by flattened epithelium and they were filled with a slightly eosinophilic cylinder and they were surrounded by lymphocytic infiltrates resembling “thyroidized areas” seen in chronic pyelonephritis (Figure 3a). Cytoplasmic granules had accumulated throughout the cytoplasm of cells in half of the PTs, and they were filled with a methenamine silver-negative, nonfluorescent proteinaceous substance. Single cells packed with cytoplasmic granules displayed apical cell membrane damage. Large calcium phosphate deposits were present at the tip of medulla.

Three normal-appearing glomeruli were observed in the semithin sections. There were PT profiles where the apical vacuoles were absent, and only a few cytoplasmic granules were seen in the darkly staining cytoplasm of cells. Other profiles had apical vacuoles, although not many, and the cells were filled with a huge number of granules containing darker or paler staining proteinaceous material (Figure 3b). The condensation of nuclear chromatin accompanied the cytoplasmic changes focally. Lastly, there were profiles containing a mixture of tubular epithelial cells without any discernable endocytic-lysosomal apparatus and cells with discernable endocytic-lysosomal apparatus.

Electron microscopically, the GBM was wrinkled focally. The podocytes were swollen, and the foot processes were segmentally effaced. On occasion, the cell bodies contained small, membrane-bound, optically clear vacuoles, and relatively large vacuoles filled with degrading material, and a few, electron-dense aggregates that were not attached to the cell membrane. The matrix of mitochondria was conspicuously dense. The rupture of vacuoles and cell membrane was found in two podocytes, and the cytosol displayed swelling and disorganization in these sites (Figure 3f). The microvilli of PTs did not have any alterations. In PT cells with no apical vacuoles light microscopically, the components of AEA could not be identified (Figure 3c). The cytosol of cells was darkly electron-dense and shrunken, and the cell organelles were tightly bunched. The very few lysosomes present did not show any digestive activity at all. The rough endoplasmic reticulum and Golgi apparatus were not discernable. In PT cells with discernible apical vacuoles in semithin sections, small and large apical vacuoles, and apical dense tubules were identified, although low in number. The cells were heavily packed with enlarged lysosomes containing homogeneous, electron-dense aggregates dispersed in a lighter but still dense matrix (Figure 3d). Other lysosomes had a rather indistinct membrane and contained much paler aggregates and the matrix was finely granular and only slightly dense (Figure 3d). The mitochondria were dysmorphic: they had various shapes, the mitochondrial matrix had an increased density, and the cristae exhibited rarefication and curved outlines (Figure 3d). Peroxisomes with crystalloid were frequently observed. Membrane-bound small vesicles had accumulated among the mitochondria, lysosomes, and peroxisomes. The cytoplasmic changes were associated in single cells with the condensation of nuclear chromatin, with or without the rupture of the apical cell membrane and cell organelles and the swelling and clearing of cell sap (Figure 3e). The ultrastructure of cells of the distal nephron was normal.

A genotypic analysis (Figure 3g) revealed DD1 affecting *CLCN5* exon 7 (c.550_551delGA/p.Asp184GlnfsTer7). The detected variant is absent in the gnomAD, Public HGMD and ClinVar database. A bioinformatical prediction supported pathogenicity since the deletion of two nucleotides might have caused a frameshift and premature STOP codon. The predicted length of the truncated mutant protein is 189 (vs. the full 818) AA length. The boy inherited the variant from his heterozygous carrier mother; and the presence of the same alteration was confirmed in his symptomatic maternal uncle’s DNA sample.

#### 2.2.3. Patient 3

A 5-year-old boy was investigated for fully blown nephrotic syndrome. Although corticosteroid treatment led to a decrease in the amount of proteinuria, it persisted and microhematuria was noted intermittently in the follow-up period. An evaluation of the renal biopsy sample led us to suspect it was a genetically induced renal disease, and the genotyping procedure confirmed it.

**Figure 4:**
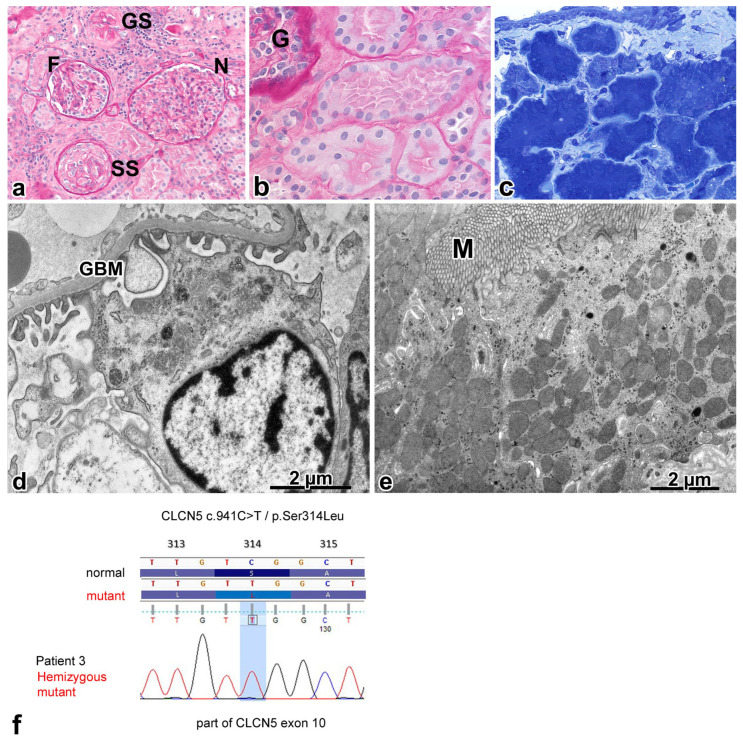
Renal biopsy alterations and genotyping results for patient 3. (**a**) A spectrum of glomerular changes: normal in appearance (N), fetal in appearance (F), globally sclerosed (GS), and segmentally sclerosed (SS) profiles; the latter displays the crescent-like proliferation of parietal cells and deposition of collagen in the urinary space (PAS, ×20). (**b**) The proximal tubular cells have no reabsorption granules in their cytoplasm. G—glomerulus (PAS, ×100). (**c**) The apical endocytic-lysosomal apparatus cannot be identified in the proximal tubular profiles shown. The cytoplasm of cells was shrunken and was stained dark blue (toluidine blue, ×63). (**d**) Part of the podocyte. The foot processes and cell organelles did not have any abnormality, and the glomerular basement membrane (GBM) was normal. (**e**) Under the microvilli (M), the apical endocytic apparatus is absent, and the lysosomes have almost disappeared. The cristae and matrix of mitochondria have not separated from each other. (**f**) A missense mutation in exon 10 of the CLCN5 gene.

Histologically, forty patent glomeruli, three globally sclerosed glomeruli, and four almost completely sclerosed glomeruli with a still discernible contour of some capillary loops and podocytes were observed. Some of the patent glomeruli had a fetal appearance (Figure 4a). There were mild foci of interstitial fibrosis and tubular atrophy, located mainly around sclerotic glomeruli. Foci of tubular calcification were noted after systematic sampling. The PT profiles had a well-developed brush border. Cytoplasmic resorption globules, typical for this nephron segment, were not observed (Figure 4b). In semithin sections, the PT profiles lacked apical vacuoles and cytoplasmic granules, the cytoplasm was dark, and the cross sections of profiles were shrunken (Figure 4c). One glomerulus displaying no change in the semithin section was examined electron microscopically. The GBM was normal. Some of the podocytes had a fetal appearance. The cell organelles and foot processes did not have any alteration (Figure 4d). As for the PT profiles, there was an absence of endocytic vacuoles and apical dense tubules under the normally appearing microvilli (Figure 4e). The very few lysosomes did not show any digestive activity at all. The cytosol of cells was shrunken, and the cell organelles were tightly bunched. The mitochondria had a homogeneous appearance, with an indistinct separation of matrix and cristae (Figure 4e). The matrix of peroxisomes was more dense than usual. The rough endoplasmic reticulum and Golgi apparatus were scarcely discernable.

The genotyping procedure (Figure 4f) detected a previously described missense variant [23] in the 10th exon of the *CLCN5* gene (NM_001127898.2:c.941C >T; NP_001121370.1:p.Ser314Leu).

**Figure 5:**
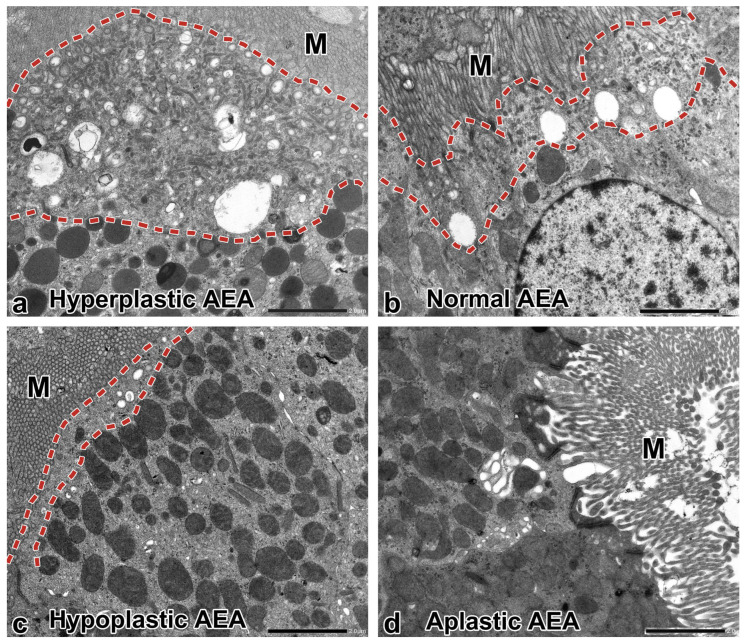
The morphologic changes of apical endocytic apparatus (AEA) at a glance. (**a**) Hyperplastic AEA. Pits at the base of microvilli (M), small and large apical vesicles, and apical dense tubules are great in number; the zone of AEA (dashed line) is markedly widened. The number of underlying lysosomes is increased. Minimal change nephropathy, ×5000. (**b**) Normal AEA. The small and large apical vesicles, the dense apical tubules, and the lysosomes are easily discernable. The zone of AEA (dashed line) appears about twice as thin as that in hyperplasia of AEA. M—microvilli. Used for illustration from a childhood case of tubulointerstitial nephritis and uveitis syndrome. ×5000. (**c**) Hypoplastic AEA. The number of apical vesicles is saliently decreased, the small and large vesicles do not differentiate from each other, and the few dense apical tubules cannot be recognized with certainty. The zone of AEA (dashed line) is significantly thinned. M—microvilli. Patient 1, ×5000. (**d**) Aplastic AEA. The small and large apical vesicles and the apical dense tubules underlying the microvilli (M) cannot be identified. Patient 2, ×5000.

**Figure 6:**
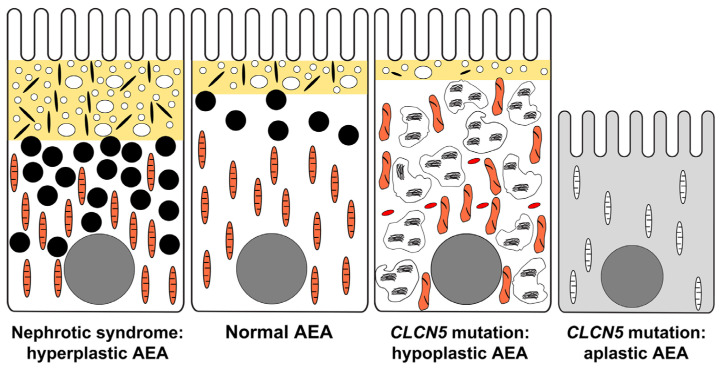
Graphical summary of alterations in apical endocytic apparatus (AEA) in normal proximal tubular ultrastructure, nephrotic range proteinuria, and pathogenic CLCN5 variant. In nephrotic range proteinuria, the zone of AEA is widened, and the number of lysosomes is increased. The mitochondria look normal, and peroxisomes with crystalloid are not seen. In pathogenic CLCN5 variant, normal-looking proximal tubular profiles alter with profiles with hypoplastic AEA or aplastic AEA. The cells with hypoplastic AEA are packed with dysmorphic lysosomes, surrounded by dysmorphic mitochondria, and increased number of peroxisomes with crystalloid (red rods). The dysmorphic lysosomes probably cannot process their protein content efficiently, and this results in lysosomal storage disease-like changes and oxidative stress-induced cell injury. Cells with aplastic AEA are shrunken and lack features of active cellular metabolism so apparent in proximal tubules adapted to endocytic demand.

Figure 5 summarizes the ultrastructural morphology of AEA in response to nephrotic conditions or pathogenic *CLCN5* variants, and Figure 6 graphically illustrates the changes of AEA and cell organelles in these conditions. Table 4 provides an at-a-glance overview of the main findings of the study.

## 3. Discussion

The indication of a renal biopsy evaluation was non-nephrotic range proteinuria. The biopsy findings ruled out some possibilities from the differential diagnosis, such as immune-complex-mediated glomerular disease and anti-tubular basement membrane nephritis [24], ultrastructural features indicative of the suspicion of Alport nephropathy, and cystinosis [16], prompting genetic testing with an expanded “FSGS panel” that included the *CLCN5* gene. Having the results of genotyping, the clinical evaluation was completed for the presence of tubular proteinuria. The supportive clinical data of DD1 in retrospect were the male gender, LMW proteinuria, hypercalciuria in patients 1 and 3, and the positive family history in the male relatives of patients 1 and 2.

Morphologically, three lesions present simultaneously characterized the *CLCN5* mutations: FGGS, alterations of the apical endocytic-lysosomal apparatus, and tubular calcific deposits. FGGS appeared as retracted, sclerotic remnants of glomeruli with or without a collagen deposition in the capsular space. The sclerotic process was sometimes only segmental, as noted by the disappearance of patency of the glomerular capillary tufts, shrinkage of GBM and mesangium admixed with the deposition of collagen, and dedifferentiation or disappearance of podocytes and adherence of such segments to parietal cells, which also displayed dedifferentiation. The degree of glomerulosclerosis was mild in patients 1 and 3, and the ultrastructural examination of podocytes did not reveal any abnormality. Patient 2 had a significant degree of glomerulosclerosis, and the patent glomeruli were enlarged; however, hyperfiltration-induced perihilar FSGS was not encountered in the biopsy sample. Electron microscopically, membrane-bound, optically clear vacuoles, vacuoles with degrading material and the electron-dense condensation of cytoplasm were seen in the podocytes, with focal ruptures of cell organelles and cell membrane. The electron-dense condensation did not involve the cell membrane, which is a typical feature of ACTN4 mutation [25]. In the light of the abnormalities observed in the PTs, discussed later, we interpreted these alterations in podocytes as the consequence of oxidative cell stress.

The alterations in PTs induced by the *CLCN5* variants were not easy to compile because Moulin et al. in 2003 [1] and Hodgin et al. in 2008 [16] could not demonstrate any ultrastructural abnormalities in PTs during the study of alltogether seven patients with DD1, which contradicted what we observed in our patients. We then came across the case study of a very rare disease, called the facio-oculo-acoustico-renal syndrome (Donnai–Barrow syndrome) in which the mutation of the LRP2 gene leads to the functional loss of megalin [26]. “Significantly less developed” AEA was observed, and this led us to think the morphological footprint of impaired endocytosis might be the hypoplasia of AEA. The recognition of hypoplastic AEA, however, requires experience. To assist training on what the AEA looked like in stimulated or decreased states of endocytosis, the luminal region of PTs appearing in resin-embedded tissue sections of biopsy samples taken from patients with heavy glomerular proteinuria and patients with DD1 was closely inspected microscopically; then corresponding areas were examined and photographed electron microscopically at fixed magnifications, and the images were placed side by side and compared. This approach allowed us to recognize the reduction or even absence of AEA in DD1 patients, discussed below.

The cases with nephrotic-range proteinuria were characterized histologically by a prominent layer of apical vacuoles and cytoplasmic protein droplets in the apical and middle part of proximal tubular cells. Electron microscopically, the zone of AEA was noticeably wide; the great number of apical dense tubules indicated an exaggerated transport of multiligand receptors to the apical membrane. The cytoplasmic protein droplets corresponded to rounded, similar-sized lysosomes, with darkly or moderately electron-dense matrix. A fraction of lysosomes displayed either electron-dense aggregates (representing most likely endocytosed and degrading LMW proteins) or membranous structures (which might have been the breakdown product of basal macroautophagy). The energy demand of the increased rate of receptor-mediated endocytosis and handling of reabsorbed substances did not affect the morphology of mitochondria, lysosomes, and peroxisomes.

The ultrastructural morphology of PTs was markedly different in the CLCN5 variants. Three types of proximal tubular cells were observed: those without alteration, those with aplastic AEA, and those with hypoplastic AEA. The distribution of normal-looking and abnormally appearing cells/PT profiles varied from visual field to field and made the recognition of *CLCN5* variant-specific changes difficult.

Cells with aplastic AEA (Figure 5d and Figure 6). The components of AEA were absent, and the lysosomes were scarce and small, and they did not have any signs of digestive activity; furthermore, it was difficult to discern the cell organelles in the shrunken cytosol. Remnants of small and large vacuoles here and there were observed in some images, but dense apical tubules were not identified despite a focused search, indicating the cessation of transport of multiligand receptors to the apical cell membrane. The appearance of cells suggested a slowing down in cellular metabolism as a consequence of severely impaired receptor-mediated endocytosis. In patient 3, almost all the PT profiles exhibited absent/rudimentary AEA in resin-embedded tissue sections.

Cells with hypoplastic AEA (Figure 5c and Figure 6). These were common in patients 1 and 2. The few apical dense tubules indicated receptor-mediated endocytosis was active at a low level. The cells were packed with dysmorphic lysosomes, and the mitochondria also did not look normal. Since the proximal tubular cells are supplied with nutrients for their cellular metabolism predominantly via apical endocytosis [27], the chronically depressed rate of receptor-mediated endocytosis might adversely affect the function of lysosomes and mitochondria, leading to disturbed crosstalk between cytoplasmic organelles [28]. The accumulation of different-sized lysosomes with electron-dense aggregates in an electron lucent matrix, and the frequent vanishing of the layering membrane indicated the lysosomes did not have the capacity to process their content properly. Histologically, the proteinaceous substance in the cytoplasmic granules did not react with methenamine silver and it was nonfluorescent in contrast to toxic lysosomal tubulopathies, e.g., calcineurin inhibitor toxicity or agrochemical toxicity, where the granules were methenamine silver-positive and displayed autofluorescence [29]. The dysmorphic features of mitochondria, namely the curved outlines of cristae, the increased density of matrix, and the variation in shape were not as conspicuous as in genetic mitochondriopathies (inclusion-like dense matrix, distended empty core, etc.) [30,31] and drug-induced tubulopathies (megamitochondria, loss of cristae, etc.) [32,33]. The dysfunction of lysosomes might induce the defective autophagy-mediated clearance of damaged mitochondria and other cytoplasmic components [34], but the small sample size and the focal distribution of PTs with hypoplastic AEA limited our retrospective morphologic investigation of the pathway. However, an increased number of apoptotic tubular cells indicating the activation of the pathway were not encountered electron microscopically. The increase in peroxisomes with crystalloid, the focal ruptures of apical cell membrane and cell organelle membrane, and the clumping of nuclear chromatin observed in patient 2 appeared to be the manifestations of oxidative stress-induced cell damage. The source of reactive oxygen species production might be the dysmorphic and presumably dysfunctional mitochondria.

## 4. Conclusions

In summary, proximal tubular cells with aplastic AEA, and proximal tubular cells with hypoplastic AEA in a patchy distribution characterized the pathogenic *CLCN5* variants. A fraction of proximal tubular cells also did not have any recognizable alterations. Since PTs with aplastic AEA and shrunken cytoplasm or PTs with hypoplastic AEA and lysosomal storage disorder-like change could not escape the attention of a person performing a focused electron microscopical evaluation, these alterations, if present, could not have been overlooked by Moulin et al. [1] or Hodgin et al. [16]. Therefore, although aplasia/hypoplasia of AEA are obvious manifestations of defective receptor-mediated endocytosis, their appearance and distribution might be influenced by the quality of the CLCN5 alteration itself. Despite this reservation, the changes of apical endocytic-lysosomal apparatus in DD1 described here for the first time in the English medical literature extend our knowledge on the morphology of the receptor-mediated endocytosis of PTs. If the proximal tubular ultrastructure has to be mentioned in the renal biopsy report, we should consider the apical dense tubules, because their presence indicates the recycling of megalin and cubilin receptors to the apical membrane, i.e., the process of receptor-mediated endocytosis is maintained (Figure 6).

## 5. Materials and Methods

The patients’ medical history and clinical data were reviewed from the clinical records. The renal biopsy specimens were evaluated via standard light microscopical stainings of formalin-fixed, paraffine embedded tissue samples, direct immunofluorescence on frozen sections (FITC-conjugated antibodies to IgG, IgA, IgM, C3, C1q, kappa, lambda, fibrinogen DakoDenmark A/S, DK-2600 Glostrup, Denmark), and electron microscopy (fixation in 3% phosphate-buffered glutaraldehyde supplemented with dextran, postfixation in 1% osmiumtetroxide, and embedding to Epon resin). The AEA and other cell organelles in PT profiles were evaluated electron microscopically in the index cases and in cases with heavy glomerular proteinuria, a condition where the activity of the megalin-cubilin endocytic receptor system was strongly stimulated [35]. Five nephrotic children (focal segmental glomerulosclerosis 2, minimal change nephropathy 2, and IgM nephropathy 1), and two adults with diabetic nephropathy and nephrotic range proteinuria were investigated. At least 10 PT profiles per case were photographed at low and higher magnifications and then compared.

### Genetic Analyses

Whole or clinical exome sequencing (WES/CES) of the three index patients was performed. Human genomic DNA was prepared from blood samples using the MagCore Genomic Whole Blood Kit (RBC Bioscience, New Taipei City, Taiwan), according to the manufacturer’s instructions. Genomic capture was carried out with either NEXTERA WES or the TrusightOne Expanded CES Kit (Illumina, San Diego, CA, USA). Massively parallel sequencing was carried out using the NextSeq500 Sequencer (Illumina, San Diego, CA, USA) in combination with the NextSeq™ 500 High Output Kit (1 × 150 bp). Raw sequence data analyses, including base calling, de-multiplexing, alignment to the hg19 human reference genome (Genome Reference Consortium GRCh37), and variant calling, were performed using an in-house bioinformatics pipeline. For variant filtration, all disease-causing variants reported in HGMD^®^, ClinVar along with all variants with minor allele frequency (MAF) of less than 1% in ExAc database were considered. Variants that possibly impaired the protein sequence, like the disruption of conserved splice sites, missense, nonsense, read-throughs, or small insertions/ deletions, were prioritized. All the relevant inheritance patterns were considered, and the candidate pathogenic mutations were verified by PCR amplification and Sanger sequencing for each individual studied.

## Figures and Tables

**Table 1:** Summary of current ultrastructural findings with previously published ultrastructural data on Dent disease type 1.

Number in Reference List	Patients/Childhood Patients Examined Ultrastructurally	Indication for Biopsy Evaluation	Electron Microscopy of Glomeruli	Electron Microscopy of Proximal Tubules
[1]	6/4	Not specified	Not mentioned	“Despite generalized PT dysfunction and LMWP, the ultrastructure of PT cells is surprisingly normal, including their tight junctions, brush border, and apical areas”
[8]	3/3	Proteinuria	FPE in 2 patients	Not mentioned
[10]	23/19	Proteinuria	Segmental FPE in 57% of patients	Focal giant mitochondria with dense condensation in 1 patient
[12]	5/1	Nephrolithiasis, renal insufficiency	Segmental FPE	Not mentioned
[13]	3/3	Proteinuria, microhematuria, tubular dysfunction	Normal	Not mentioned
[14]	1/1	Proteinuria, microhematuria, tubular dysfunction	Normal	Not mentioned
[15]	1/1	Superimposed glomerular disease on Dent disease?	Mesangial electron-dense deposits of C1q nephropathy	Not mentioned
[16]	1/1	Proteinuria, glycosuria	Minimal FPE	Well-preserved brush border, normal tubular basement membranes
[17]	3/3	Proteinuria; in nephrotic range in 2 patients	Segmental FPE in 1 patient	Not mentioned
[18]	1/0	Proteinuria, microhematuria, nephrolithiasis	No change	Not mentioned
[19]	5/5	Nephrotic-range proteinuria	Segmental FPE	Not mentioned
[20]	5/4 (1 rebiopsy)	Variable; nephrotic-range proteinuria in 2 patients	Segmental FPE in 3 patients	Not mentioned
[21]	1/1	Proteinuria	Normal	Not mentioned
[22]	1/1	Proteinuria, microhematuria; psoriasis	Electron-dense deposits of C3 glomerulonephritis	Not mentioned
Present publication	3/3	Non-nephrotic proteinuria	Segmental FPE in 2 patients, podocyte cell body abnormality in 1 patient	Focal hypoplasia/aplasia of AEA; consequences of impaired endocytosis in lysosomes, mitochondria, and peroxisomes

Abbreviations: AEA—apical endocytic apparatus, C1q—complement factor 1q, C3—complement factor 3, FPE—foot process effacement, LMWP—low molecular weight proteinuria, PT—proximal tubular.

**Table 2:** Clinical characteristics of male patients with Dent disease type 1 at the time of the kidney biopsy.

Boy Patient	Age y	Protein-Uria g/d	ß-2-MG	Micro- Hematuria	Hyper-Calciuria	Glycos- Uria	Phosphat- Uria	eGFR mL/min/1.73 m^2^	Renal Ultrasound	Family History in Male Relatives
1.	6.5	1	**↑↑↑**	Intermittent	Yes	No	No	86	No change	Brother affected
2.	13.5	2.9	**↑↑↑**	No	No	No	No	66	Smaller kidneys, punctate lesions suspicious for nephrocalcinosis	Uncle affected
3.	5	0.75	**↑↑**	Intermittent	Yes	No	No	124	No change	Negative

Abbreviations: ß-2-MG: ß-2-microglobulinuria, DD1: Dent disease type 1, **↑**—grade of ß-2-microglobulinuria, eGFR: estimated glomerular filtration rate, g/d: gram/day, y: year.

**Table 3:** Kidney biopsy findings in patients with Dent disease type 1.

Patient	*CLCN5* Mutation	FGGS %	FSGS %	Enlarged Glomeruli	Fetal Glomeruli	IFTA %	Chr. PN-like Thyroidized Areas	Tubular Calcium Deposits	Podocyte FPE %	Podocyte Cell Body Abnormality	PTs with Aplastic AEA *	PTs with Hypoplastic AEA **
1.	Frameshift	13.6	4.5	No	No	0	No	Yes	20	No	Several	Few
2.	Frameshift	33.6	0	Yes	No	25	Yes	Yes	20	Yes	Few	Several
3.	Missense	6.3	8.5	No	Yes	10	No	Yes	0	No	Almost all	None

* Absent or rudimentary apical endocytotic apparatus, the absent or markedly decreased number of lysosomes, mitochondrial cristae difficult to discern, shrunken cytosol. ** A narrow zone of apical endocytotic apparatus, the accumulation of dysmorphic lysosomes storing proteinaceous material, dysmorphic mitochondria; an oxidative stress-like cellular injury in patient 2. In each patient, the arteries and arterioles were unremarkable, and the immunofluorescent evaluation did not reveal any specific staining. The apoptosis of proximal tubular epithelial cells was not encountered with an ultrastructural examination. Abbreviations: AEA: apical endocytic apparatus, Chr. PN: chronic pyelonephritis, FGGS: focal global glomerulosclerosis, FPE: foot process effacement, FSGS: focal segmental glomerulosclerosis, IFTA: interstitial fibrosis and tubular atrophy, PT: proximal tubule.

**Table 4:** Overview of the genetic, clinical, and renal biopsy alterations observed in pathogenic *CLCN5* variants.

Boy; Age (y)	Patient 1; 6.5 y	Patient 2; 13.5 y	Patient 3; 5 y
Pathogenic *CLCN5* variant	Frameshift, not yet described	Frameshift, not yet described	Missense, already described
Renal ultrasound	No change	Smaller kidneys, nephrocalcinosis in suspicion	No change
Proteinuria	Yes	Yes	Yes
Microhematuria	Intermittent	No	Intermittent
ß-2-microglobulinuria	Yes	Yes	Yes
Hypercalciuria	Yes	No	Yes
Glycosuria/phosphaturia	No	No	No
History of renal disease in male relatives	Yes	Yes	No
*Light microscopical alterations*			
Focal global GS	Yes	Yes	Yes
Focal segmental GS	Yes	No	Yes
Interstitial fibrosis and tubular atrophy	No	Yes	Yes
Chronic pyelonephritis-like areas	No	Yes	No
Calcium deposits	Yes	Yes	Yes
Lysosomal storage of proteins in PTs	Yes, occasional	Yes, conspicuous	No
*Electron microscopical alterations*			
Podocyte cell body abnormalities	No	Yes	No
GBM	Normal	Normal	Normal
Foot process effacement	Segmental	Segmental	No
PTs with hypoplastic AEA	Few	Several	None
Dysmorphic lysosomes	Yes	Yes, features of storage disease	No
Dysmorphic mitochondria	Yes	Yes	No
Peroxisomes with inclusion	Yes	Yes	No
Oxidative stress-like injury in PT cells	Not observed	Yes	No
PTs with aplastic AEA	Several	Few	Almost all
Disappearance of lysosomes	Yes	Yes	Yes
Inactive cell organelles	Yes	Yes	Yes
Shrinkage of cells	Yes	Yes	Yes

Male gender, proteinuria, ß-2-microglobulinuria, focal global glomerulosclerosis, distinct abnormalities of apical endocytic-lysosomal apparatus, and calcium deposits were characteristic of Dent 1 disease at the time of kidney biopsy evaluation. Abbreviations: AEA—apical endocytic apparatus, GBM—glomerular basement membrane, GS—glomerulosclerosis, PT—proximal tubular, PTs—proximal tubules, y—year.

## Data Availability

Sequence data is unavailable due to privacy and ethical restriction.
